# Supplementary material for: Health care professionals intention to use digital health data hub working in East Gojjam Hospitals, Northwest Ethiopia: Technology acceptance modeling
Source: PLoS One. 2025 May 15;20(5):e0322460. doi: 10.1371/journal.pone.0322460 (PMC12080794; doi:10.1371/journal.pone.0322460)
Supplement: S1 Appendix — (DOCX) [file pone.0322460.s001.docx]

# Annex

# Information sheet

**Title:** Health care professional intention to use digital health Hub working at East Gojjam Hospital in North West, Ethiopia: Technology acceptance modeling.

**Introduction**: The study was made by a research investigator to determine Healthcare professionals’ intention to use digital health hubs working at East Gojjam Hospital in North West, Ethiopia in 2024.

**Principal Investigator:** Ayenew Sisay (MPH in Health Informatics)

**Co-Investigators:**

1. Mr. Bayu Tilahun (MPH in Health Informatics)
2. Mr. Afework Edmealem (MSC in Nursing)
3. Mr. Habtamu Mekonen (MSC in Human Nutrition)
4. Mrs. Tirsit Ketsela(MSC in Pharamcy)
5. Mr. Melese Getachew (MSC in Pharamcy)
6. Mr. Andualem Fentahun (MPH in Health Informatics)
7. Mr. Sefefe Birhanu (MPH in Health Informatics)

**Purpose**: - The study aims to assess healthcare professionals' intentions to use digital health hubs. The findings help to strengthen the users who are working in hospitals to improve digital health data hub culture and use (DH*2) to help make informed decisions about health care activities.

**Procedure**: A hospital-based cross-sectional study design was applied to health professions in East Gojjam Hospital in North West, Ethiopia, and the data was collected from 2024 and then analysed using STATA software.

**Risk and confidentiality**: The study dose not pose any risk to the study participant, and I assure you that I keep the information confidential and only use it for academic purposes.

## Consent form statement

Code Number -----/------

Hospital __________________________________

Greetings, Sir/Madam

Hello, my name is_____. I am working for Debre Markos University as an instructor in the health care informatics department. Now, I plan to conduct the research entitled **“**Health care professional intention to use digital health hub working at East Gojjam Hospital in North West Ethiopia."

Dear honor, I am happy to dedicate you to this research by completing the following survey questions: Before you make a decision, you should know why the research is being conducted and what it will entail for you. Please inquire if there is anything unclear or if you require additional information.

The study aims to assess the intention to use digital health data hubs in healthcare among healthcare professionals at East Gojjam hospitals. The findings will help to strengthen health professionals' use of digital health data hubs while working in hospitals because it enhances the digital health data culture and supports to use right health care data for the right decision. It is absolutely your decision whether you would like to participate or not. If you are willing to participate after reading this information sheet, please fill in the questions neatly. You can complete the study freely or leave at any moment without giving a reason. This questionnaire will require approximately 5 minutes of your cooperation. Finally, I assure you that I will keep the information confidential and only use it for academic purposes.

B. Consent Certificate

Firstly, I have access to electronic health data, and I understand the outcomes of this study. This study result will be important for the development of digital health information transformation. I hope the study will be distributed to healthcare providers, the health information administration, and the Minister of Health.

I am voluntarily taking part in this research.

I agree I disagree

Sign ________________________

Thank you in advance!

| Part I: Screening questions |
| --- |
| - - - 1. Have you used a digital personal device in the workplace? - Yes - No |
| - - - 1. Which electronic devices are mostly used? - Smartphone - Digital computer (laptop, desktop, tablet or others |
| - - - 1. How often have you used online digital personal data stores in your work settings? - Never - Seldom - Sometimes - Often - Almost Always |
| - - - 1. Which digital data store is the most preferred? - Dropbox - OneDrive - Google Drive - Hub of All Things - Apple iCloud - Databox - Digime - Other, specify _______ - Never pay most preferred |

| **Questionnaire** Part I: Sociodemographic characteristics | | |
| --- | --- | --- |
| 1. Institution Name: ________________ | | |
| 1. Gender | - Male | - Female |
| 1. Age ___________ year | | |
| 1. Religion | - - Orthodox   - Muslim   - Catholic | - - Protestant   - Other |
| 1. Marital status | - Single - Married - Separated | - Divorced - Widowed |
| 1. Professions | - - Physician - Pharmacy - Anastasia - Health officer - Optometry   - Medical laboratory | - - Radiologist   - Health information   - Nurse   - Midwives   - Physiotherapy   - Psychiatry |
| 1. Educational status | - Bachelor degree - Master’s degree - Specialist - Subspecialist | - Assistant professor - Associate professor - Professor - Others |
| 1. Work experience: ______________ year | | |
| 1. Monthly salary: _________________ birr | | |

Part III: Digital Health Data Hub questions

| **BI: Behavioural Intention** | 1 | 2 | 3 | 4 | 5 |
| --- | --- | --- | --- | --- | --- |
| BI1: I will intend to use digital health data store or hub |  |  |  |  |  |
| BI2: I will plan to use digital health data hub |  |  |  |  |  |
| BI3: I will continue to use digital health data hub |  |  |  |  |  |
| BI4: I will inform other of the goodness of digital health data hub to use |  |  |  |  |  |

| **PU**: **Perceived Usefulness** | 1 | 2 | 3 | 4 | 5 |
| --- | --- | --- | --- | --- | --- |
| PU1: Digital health data hub enables me to store my personal data how I want to. |  |  |  |  |  |
| PU2: Digital health data hub enhances my effectiveness in storing health data (ability to accomplish the necessary tasks to store data) |  |  |  |  |  |
| PU3: Digital health data hub makes it easier to store health data |  |  |  |  |  |
| PU4: Digital health data hub increases productivity in storing health data (the time need to do the tasks related to storing data) |  |  |  |  |  |
| PU5: I find digital health data hub to be useful in my day-to-day life |  |  |  |  |  |

| **PEOU: Perceived Ease of Use** | 1 | 2 | 3 | 4 | 5 |
| --- | --- | --- | --- | --- | --- |
| PEOU1: Interacting with digital health data hub may not require a lot of my mental effort |  |  |  |  |  |
| PEOU2: I find digital health data hub to be easy to use |  |  |  |  |  |
| PEOU3: I find it easy to get digital health data hub to do what I want them to do |  |  |  |  |  |

| **PT: Perceived Trust** | 1 | 2 | 3 | 4 | 5 |
| --- | --- | --- | --- | --- | --- |
| PT1: I perceive that using digital health data hubs are dependable |  |  |  |  |  |
| PT2: I trust in the ability of digital health data hubs to safeguard my personal data |  |  |  |  |  |
| PT3: I strongly believe that Digital health data hubs are trustworthy |  |  |  |  |  |

| **ATT: Attitude toward Use** | 1 | 2 | 3 | 4 | 5 |
| --- | --- | --- | --- | --- | --- |
| ATT1**:** I will use the digital health data hubs only if I can utilize it |  |  |  |  |  |
| ATT2: I will use the digital health data hubs if I find it rewarding to use |  |  |  |  |  |
| ATT3: I will use a digital health data hub to store personal data if it is reasonably priced |  |  |  |  |  |

| **Perceived Risk** | 1 | 2 | 3 | 4 | 5 |
| --- | --- | --- | --- | --- | --- |
| PR1: Digital health data hubs may not provide adequate protection of personal health data |  |  |  |  |  |
| PR2: Digital health data hubs may not protect the privacy of its users |  |  |  |  |  |
| PR3: Digital health data hubs will share personal health information without authorization |  |  |  |  |  |

If you have any suggestion, write here.

____________________________________________________________________________________________________________________________________________________________________________________________________________________________________________________________________________________

Thank you so much for your participation!
